# Gynaecological endoscopic surgical education and assessment. A diploma programme in gynaecological endoscopic surgery

**DOI:** 10.1007/s10397-016-0957-1

**Published:** 2016-06-21

**Authors:** Rudi Campo, Arnaud Wattiez, Vasilis Tanos, Attilio Di Spiezio Sardo, Grigoris Grimbizis, Diethelm Wallwiener, Sara Brucker, Marco Puga, Roger Molinas, Peter O’Donovan, Jan Deprest, Yves Van Belle, Ann Lissens, Anja Herrmann, Mahmood Tahir, Chiara Benedetto, Igno Siebert, Benoit Rabischong, Rudy Leon De Wilde

**Affiliations:** Life Expert Centre, Schipvaartstraat 2 Bus 4, 3000 Leuven, Belgium; European Academy for Gynaecological Surgery, Diestsevest 43/0001, 3000 Leuven, Belgium; European Society for Gynaecological Endoscopy, Diestsevest 43/0001, 3000 Leuven, Belgium; European Board and College of Obstetrics and Gynaecology, Brussels, Belgium; Department of Women’s Health, University Hospital Tuebingen, Calwerstraat 7, 72077 Tuebingen, Germany; Center for Surgical Technologies, Leuven, Belgium; University Hospitals Leuven, Leuven, Belgium; Pius-Hospital Oldenburg, Department of Gynecology, Obstetrics and Gynaecological Oncology, Carlvon Ossietzky University, Georgstraße 12, 26121 Oldenburg, Germany; African Endoscopic Training Academy, Cape Town, South Africa; International Centre for Endoscopic Surgery, Clermont-Ferrand, France

**Keywords:** Laparoscopy, Hysteroscopy, Practical skills, Education, Endoscopic surgery

## Abstract

In recent years, training and education in endoscopic surgery has been critically reviewed. Clinicians, both surgeons as gynaecologist who perform endoscopic surgery without proper training of the specific psychomotor skills, are at higher risk to increased patient morbidity and mortality. Although the apprentice-tutor model has long been a successful approach for training of surgeons, recently, clinicians have recognised that endoscopic surgery requires an important training phase outside the operating theatre. The Gynaecological Endoscopic Surgical Education and Assessment programme (GESEA) recognises the necessity of this structured approach and implements two separated stages in its learning strategy. In the first stage, a skill certificate on theoretical knowledge and specific practical psychomotor skills is acquired through a high-stake exam; in the second stage, a clinical programme is completed to achieve surgical competence and receive the corresponding diploma. Three diplomas can be awarded: (a) the Bachelor in Endoscopy, (b) the Minimally Invasive Gynaecological Surgeon (MIGS) and (c) the Master level. The Master level is sub-divided into two separate diplomas: the Master in Laparoscopic Pelvic Surgery and the Master in Hysteroscopy. The complexity of modern surgery has increased the demands and challenges to surgical education and the quality control. This programme is based on the best available scientific evidence, and it counteracts the problem of the traditional surgical apprentice-tutor model. It is seen as a major step toward standardisation of endoscopic surgical training in general.

## Introduction

In recent years, training and education in endoscopic surgery has been critically reviewed [1, 2]. Laparoscopy has gained wider acceptance within the surgical community as a preferred tool and became the golden standard, instead of laparotomy, for diagnosis and treatment of many diseases [1, 2]. Laparoscopic procedures provide higher surgical competence and improved patient outcome [3, 4] coherent with a reduction in blood loss, postoperative pain, infection rates and hospital stays [5, 6]. However, laparoscopic procedures are not commonly applied in complex procedures, because only a minority of surgeons possess advanced laparoscopic skills [7].

An endoscopic surgeon ideally must possess theoretical background of anatomy, pathology, treatment options, surgical techniques and adequate practical laparoscopic psychomotor skills (LPS) [8], including laparoscopic camera navigation (LCN), hand-eye coordination (HEC) and bi-manual coordination (BMC), prior to enter the in-operating room (OR) training programme. Laparoscopic skills are difficult to learn. In particular, laparoscopy requires excellent HEC on a 2D screen and counterintuitive movements for manipulating instruments [2].

Surgical competence can only be acquired if the in-OR teaching is performed by a highly skilled surgeon and is characterised by a continuous learning process. The apprentice first observes the procedure then assists the surgeon and finally operates under guidance. However, in endoscopic and more specific in laparoscopic surgery, the surgical training must be preceded by structured dry skill lap training with the acquisition of the specific LPS. The learning characteristics of LPS in contrary to the surgical competence do not require constant supervision from a highly skilled surgeon but relies on repetitive practise, and once gained, these abilities are retained over a long period of time (unpublished observations) [9–13].

Clinicians, both surgeons as gynaecologist who perform endoscopic surgery without proper training of the specific psychomotor skills, are at higher risk to increased patient morbidity and mortality [14–16]. Although the apprentice-tutor model has long been a successful approach for training of surgeons, recently, clinicians have concluded that endoscopic surgery requires an important training phase outside the operating theatre.

**Fig. 1:**
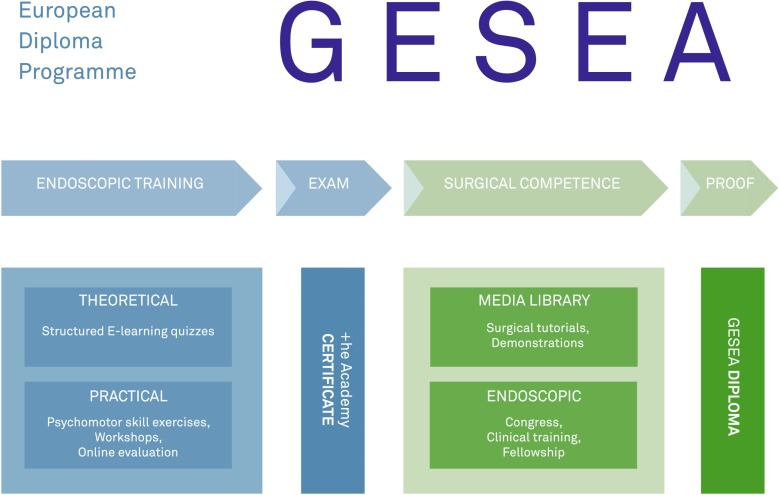
The Gynaecological Endoscopic Surgical Education and Assessment (GESEA) structured learning and validation path

The Gynaecological Endoscopic Surgical Education and Assessment (GESEA) recognises the necessity of this structured approach and implements two assessment stages in its learning strategy. In the first stage, a skill certificate on theoretical knowledge and specific practical psychomotor skills is acquired through a high-stake exam; in the second stage, a clinical programme is completed to achieve surgical competence and receive the corresponding diploma (Fig. 1) [17–23].

**Fig. 2:**
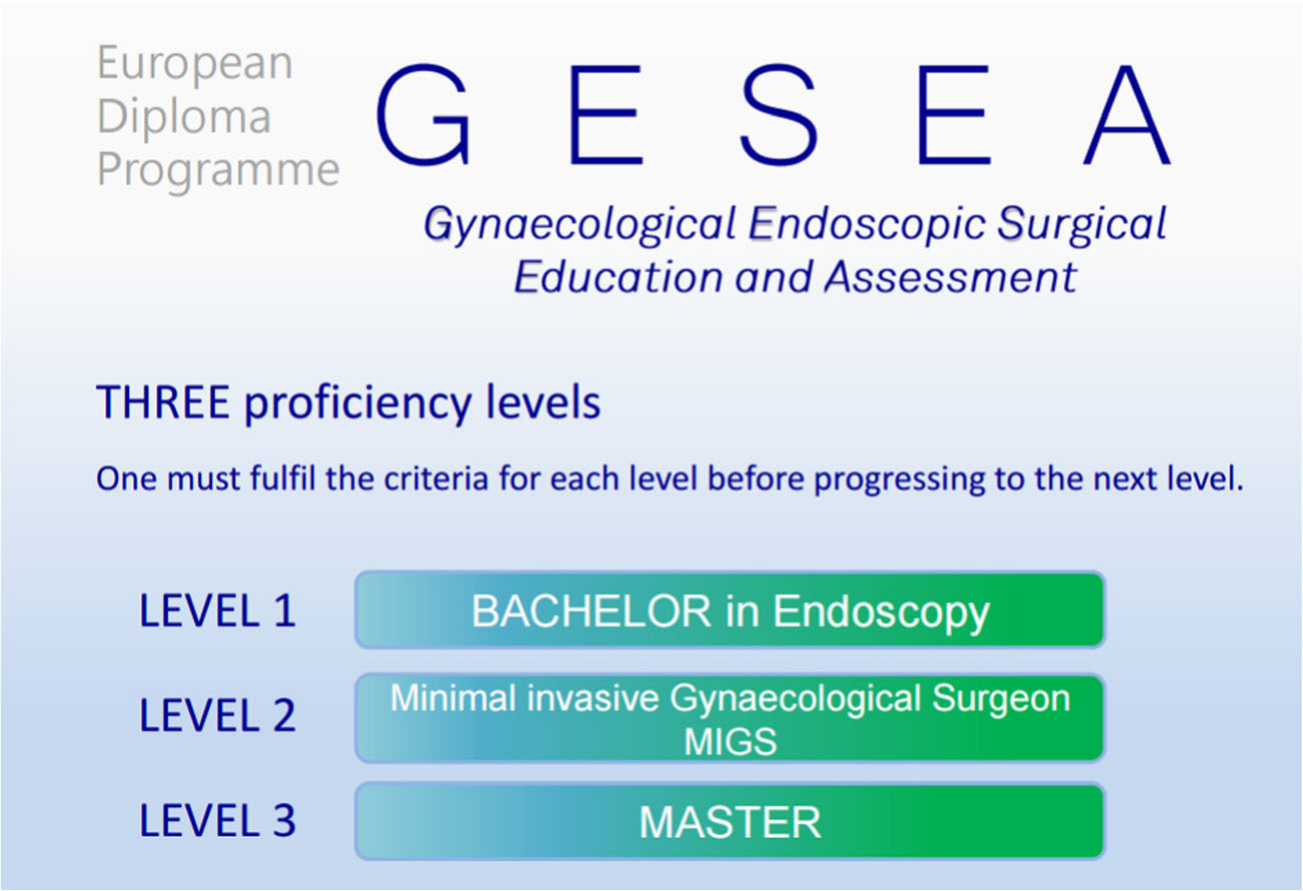
The three proficiency levels of the GESEA programme

Three diplomas can be awarded: (a) the Bachelor in Endoscopy, (b) the Minimally Invasive Gynaecological Surgeon (MIGS) and (c) the Master level. The Master level is sub-divided into two separate diplomas: the Master in Laparoscopic Pelvic Surgery and the Master in Hysteroscopy (Fig. 2) [17–23].

The European Society for Gynaecological Endoscopy (ESGE) is responsible for the diploma in collaboration with the European Board and College of Obstetrics and Gynaecology (EBCOG) [21]. +he Academy is the notified body for the high-stake exam and for issuing +he Academy skill certificate (Fig. 1) [20].

The complexity of modern surgery has increased the demands and challenges to surgical education and the quality control. This programme is based on the best available scientific evidence, and it counteracts the problem of the traditional surgical apprentice-tutor model. It is seen as a major step toward standardisation of endoscopic surgical training in general.

## Training

Prior to enter the in-OR training, a theoretical and practical programme with self-evaluation modules is defined.

An online teaching programme is provided to train and test the theoretical knowledge (www.websurg.com/winners/). This programme offers a set of peer-reviewed tutorials and the possibility of self-assessment by means of five multiple choice questions (MCQ’s) randomly chosen from a pool after each tutorial section. When these MCQ’s are correctly answered, then the topic is approved. As the MCQ’s are not correctly answered, a new set of five MCQ’s is provided [24–29]. Only when all topics for a specific level have been passed, then the participant can be considered as a candidate for +he Academy certification.

+he Academy has developed a series of tools and methods for training and testing of practical endoscopic skills: the Laparoscopic Skills Training and Testing model (LASTT), the Suturing Training and Testing model (SUTT) and the Hysteroscopic Training and Testing model (HYSTT) [21].

The LASTT model can be used as an insert in a conventional trainer box and comprises three different exercises that aim to train and evaluate three specific LPS: LCN, HEC and BMC. The result of an exercise is expressed in time to correct performed exercise [18]. Construct, content and face validity of those exercises have been published [30, 31].

The SUTT model has been developed to train and test more complex and fine LPS like needle manipulation, intracorporeal knotting, cutting and tissue approximation using both dominant and non-dominant hands. These exercises are performed in a pelvic trainer with a 0° 10-mm optic and two needle holders.

The HYSTT model represents the spatial distribution and orientation of the different planes and angles of a normal uterus. Here a 2.9-mm 30° optic is used and two exercises are defined to train and test camera navigation and HEC.

The results of each exercise are reported on an online scoring platform providing the surgeon his position in the benchmark population and an allocation to the excellent, fair and room for improvement group.

## Certificate

To validate the knowledge and endoscopic practical psychomotor skills, +he Academy has developed a high-stake exam.

The theoretical exam consists of 50 MCQ’s to evaluate the knowledge of the individual in the specific areas of expertise according to the level. The practical exam consists of the three LASTT exercises, the level corresponding SUTT and HYSTT exercises, which are performed in a standardised environment supervised by a director of examination and one accredited mentor for each working station.

The exam is performed at international congresses and in an accredited GESEA diploma centre. Within 14 days, the participant receives the global result as a pass, by receiving +he Academy skill certificate, or a fail (Fig. 1). No detailed information as regards the scores of the different tests is provided. If the mentee fails the exam, then the total exam has to be repeated. In case of dispute, the mentee can address a complaint to the exam appeal commission.

## Diploma of surgical competence

Each level of the GESEA curriculum results in a diploma (Fig. 1); the Bachelor, Minimal Invasive Gynaecological surgeon and Master diploma (Fig. 2).

The bachelor diploma, specifically designed for residents or endoscopists who carried out less than 200 interventions, can be viewed as a prerequisite to starting the in-OR clinical training in endoscopic surgery. Requirements for this diploma are +he Academy Bachelor skill certificate, exposure as an observer to at least 30 endoscopic procedures and proof of attendance of a recognised endoscopic congress or workshop.

Requirements for the MIGS diploma are +he Academy MIGS skill certificate, proof of a predefined surgical clinical curriculum in laparoscopy and hysteroscopy in a period of max. 5 years, 50 CME/CPD points of endoscopic congresses or workshops and 20 ESGE educational points including scientific contribution (e.g. publication), mentorship, etc.

The Master diploma can be achieved separately for laparoscopy and hysteroscopy, which follows the same flow chart as the MIGS diploma.

## Conclusion

The endoscopic approach to surgical patient care has a different dimension in the learning process in comparison to the traditional ‘open’ surgery. The specialised equipment and instrumentation require a different set of technical skills and organisation of the surgical team [28].

Professional organisations are responsible for setting the standards for training the next generation of specialists to ensure patient safety. The training programme should be standardised, include objective metrics of validation, offer universal accessibility and provide credentials to confirm successful training [23–28].

The innovative approach of the GESEA programme has acknowledged the need for different skills with different learning paths. Surgical knowledge and practical skill performance are evaluated with objective methods. However, the criteria for acquiring the GESEA diplomas are related to performance, continuous medical education and professional development.

The GESEA programme follows minimal standards and provides a structured training path for the endoscopy surgeons. This programme provides training in endoscopic procedures with built-in safety and the best possible surgical outcome. GESEA criteria increase the quality of the one-to-one clinical training programme in endoscopic procedures for all stakeholders [17–23].

Full article is available on www.ebcog.org and www.esge.org.
